# Plasma Interleukin-10 Levels Are Altered in Women with Severe Premenstrual Syndrome: A Preliminary Study

**DOI:** 10.1089/whr.2019.0010

**Published:** 2020-03-04

**Authors:** Kaori Yama, Yuki Asari, Aiko Ono, Maiko Machida, Jun Miura

**Affiliations:** Faculty of Pharmaceutical Sciences, Hokkaido University of Science, Sapporo, Japan.

**Keywords:** depression, inflammation, interleukin-10, premenstrual syndrome

## Abstract

***Background:*** The precise pathophysiology of premenstrual syndrome (PMS) is unknown, and chronic inflammation has been implicated in PMS. However, inflammatory markers, including cytokines and C-reactive protein (CRP), have not been investigated before and after menstruation in relation to PMS among the same participants. This study investigated whether the plasma levels of tumor necrosis factor-α, interleukin (IL)-6, IL-10, and CRP are related to PMS.

***Methods:*** The study included 21 healthy Japanese women (aged 19–24 years) with a regular menstrual cycle. Inflammatory marker levels in plasma were determined using enzyme-linked immunosorbent assay. In addition, the level of depressiveness was assessed using the Center for Epidemiologic Studies Depression (CES-D) scale.

***Results:*** Of the 21 women, 7 were considered to have moderate-to-severe PMS (PMS [+] group) and 14 were considered to have no or mild PMS (PMS [−]), and none of the participants had premenstrual dysphoric disorder. The IL-10 levels were significantly lower before than after menstruation in the PMS (−) group. The IL-10 levels before menstruation were significantly higher in the PMS (+) group than in the PMS (−) group. Other markers did not show relevant differences between the groups. The CES-D scores were higher in the PMS (+) group than in the PMS (−) group both before and after menstruation. There were positive correlations between the CES-D scores and IL-6 levels before menstruation and the CES-D scores and IL-10 levels after menstruation.

***Conclusions:*** The IL-10 levels before menstruation were higher in women with PMS than in those without PMS, and these levels might be related to PMS.

## Introduction

Population-based studies have suggested that 30%–40% of reproductive-age women meet the clinical criteria for premenstrual syndrome (PMS).^1,2^ According to ICD-10, PMS is characterized by symptoms, such as depression, anxiety, irritability, fatigue, breast tenderness, and sleep disturbance, which occur 1–2 weeks before menstruation and disappear after the start of menstruation.^3^ PMS has been shown to negatively influence relationships, work attendance, productivity, and health care cost and utilization.^1^ The precise pathophysiology of PMS is still unknown, but several possible causes have been suggested, including decreased levels of progesterone, the neurotransmitters serotonin, and gamma-aminobutyric acid. The pathogenesis of PMS should be elucidated, and effective treatments for PMS should be established.

Chronic inflammation has been implicated in the etiologies of depression and other psychiatric and somatic disorders that share common features with PMS.^4,5^ Cytokine expression is observed in the endometrium, ovarian tissue, and granulosa cells.^6–8^ Cytokines are believed to play roles in leukocyte recruitment, vascularization, and tissue remodeling and repair during the menstrual cycle.^9^ In healthy women with a normal menstrual cycle, the levels of plasma and endometrial inflammatory markers, including C-reactive protein (CRP) and the cytokines interleukin (IL)-6, IL-1β, and tumor necrosis factor (TNF)-α, have been shown to vary during the menstrual cycle.^7,10–15^ For example, Whitcomb et al. performed a longitudinal study of 250 women with a normal menstrual cycle and found significant variability in the plasma levels of cytokines, including IL-1β, IL-8, and IL-10.^15^ The levels of several inflammatory factors have been shown to increase around the time of ovulation and later peak during menstruation, which is considered a proinflammatory event.^16,17^ The levels of IL-1β, IL-6, and IL-8 have been shown to inversely correlate with the levels of estradiol and progesterone, further supporting immune involvement in normal menstrual cycle function.^15^ In addition, a previous study of 15 healthy women found that CRP levels were positively related to menstrual symptom severity, especially regarding mood and pain symptoms.^12^ However, as women with PMS were excluded from these studies, the associations of inflammation with severe menstrual symptoms and PMS remain largely unclear. Bertone-Johnson et al. mentioned that inflammatory markers might be elevated in women experiencing PMS.^18^ However, in this previous study, all samples were obtained in the mid-luteal phase of the menstrual cycle. To our knowledge, inflammatory markers, including cytokines and CRP, have not been investigated before and after menstruation in relation to PMS among the same participants.

This study aimed to investigate whether the plasma levels of TNF-α, IL-6, IL-10, and CRP (markers of inflammation) are associated with premenstrual symptoms.

## Materials and Methods

### Participants

This study was conducted from February 2017 to January 2018. Participants were recruited from among students of the Hokkaido University of Science. All participants were healthy Japanese women with a regular menstrual cycle. They were not taking any medications, supplements, or health foods, they did not smoke, and they had no medical history of serious illnesses. Participants with abnormalities of hemostatic function and coagulability were excluded.

The study enrolled 32 healthy Japanese women aged 19–24 years. To confirm the luteal phase, progesterone levels were measured by electrochemiluminescence immunoassay (SRL, Inc., Sapporo, Japan). The reference progesterone level for confirming the luteal phase, as suggested by SRL, Inc., was >2.05 ng/mL. Participants with progesterone levels below this reference were excluded from the analysis.

All participants provided written informed consent after receiving explanations of the purpose and design of this study. The study was approved by the Ethics Committee of Hokkaido Pharmaceutical University (No. 16-02-008) and was carried out in accordance with the ethical principles of the Declaration of Helsinki.

### Assessment of PMS

Psychological and physical symptoms before menstruation were assessed using the premenstrual dysphoric disorder (PMDD) scale developed by Miyaoka et al.^19^ by modifying the premenstrual symptoms screening tool developed by Steiner et al.^20^ The PMDD scale includes the following two parts: Section A, which refers to symptoms, and Section B, which refers to interference with activities or relationships. Section A includes questions on depressive mood, anxiety, tearfulness, anger, decreased interest, concentration difficulty, fatigability, overeating, hypersomnia, insomnia, feeling out of control, and physical symptoms. Section B includes questions on interference with efficiency at work or school, housework, coworker relationships, family relationships, and friend relationships. The severity of symptoms or interference was rated as “not at all,” “mild,” “moderate,” or “severe.” Participants were considered to have moderate-to-severe PMS if they answered “moderate” or “severe” for at least five items in section A (at least one of which was within the first four items) and answered “moderate” or “severe” for at least one of the items in section B. Participants with other responses were considered to have no or mild PMS.

### Assessment of inflammatory markers

Whole blood (10 mL) was collected into a heparin tube and immediately centrifuged at 1200 rpm in a refrigerated centrifuge at 4°C for 10 min to prepare plasma samples. The samples were stored at −80°C until analysis. Samples were blinded with regard to participant characteristics and were assayed for the levels of TNF-α, IL-6, IL-10, and CRP. The plasma cytokine and CRP levels were determined using enzyme-linked immunosorbent assay (ELISA). Human TNF-α, IL-6, and IL-10 Quantikine ELISA Kits (R&D Systems, Inc., Minneapolis, MN) were used to assess TNF-α, IL-6, and IL-10, and the CircuLex High-Sensitivity CRP ELISA Kit (CycLex, Co., Ltd., Nagano, Japan) was used to assess CRP. Measurements were made according to the manufacturer protocols. Samples were assessed in duplicate with internal standards. Calibration curves were prepared using the internal standards, and the plasma cytokine and CRP levels were calculated using the calibration curves. The cytokine and CRP results are expressed in pg/mL and mg/L, and the limits of quantitation for TNF-α, IL-6, IL-10, and CRP were 5.5, 3.1, 7.8, and 59.4 pg/mL, respectively.

### Assessment of depression

The level of depressiveness was assessed using the Center for Epidemiologic Studies Depression (CES-D) scale.^21^ This assessment was performed before and after menstruation. The CES-D scale is a four-grade (0–3) scale for the self-assessment of depression, and it includes 20 question items. The Japanese version of the CES-D scale was used in this study.^22^ The total score ranges from 0 to 60, and a higher score indicates being more depressive.^22^

### Statistical analysis

Demographic characteristics, including age, menstrual cycle, and timing of blood sample collection, were compared between participants with and those without PMS using the Student's *t*-test. Data are presented as median (interquartile range [IQR]). The Wilcoxon signed-rank test was used to compare median values before and after menstruation. The Mann–Whitney *U* test was used to compare differences between participants with and those without PMS. The correlation of each inflammatory marker and the CES-D score was analyzed using Spearman's rank correlation coefficient, in each phase. The significance level was set at 0.05. All analyses were performed using the statistical software Bell Curve Excel statistics for Windows (Social Survey Research Information Co., Ltd., Tokyo, Japan).

## Results

### Demographic characteristics of the study participants

Blood samples were collected from 32 participants; however, the progesterone levels in 11 participants were not within the reference for the luteal phase. Thus, the samples of the remaining 21 participants aged 19–24 years (median [IQR] age, 22 [21, 22] years) were analyzed in this study. The median menstrual cycle duration was 29.0 (28.0, 31.5) days. Overall, blood samples were collected a median of 4.0 (5.0, 2.0) days before the start of menstruation and a median of 7.5 (6.0, 7.0) days after the beginning of menstruation. The median progesterone level before menstruation was 7.9 (4.5, 12.5) ng/mL. Of the 21 participants, 7 were considered to have moderate-to-severe PMS (PMS [+] group) and 14 were considered to have no or mild PMS (PMS [−] group), and none of the participant had PMDD. Because none of the participants had PMDD, participants having severe-to-moderate PMS except for PMDD were categorized as PMS (+).

The median ages of the participants in the PMS (+) and PMS (−) groups were 21 (20, 22) years and 22 (22, 22) years, respectively. In addition, the median menstrual cycle durations in the PMS (+) and PMS (−) groups were 28.5 (27.8, 30.5) days and 30.0 (28.0, 31.5) days, respectively. Moreover, the median progesterone levels before menstruation in the PMS (+) and PMS (−) groups were 8.3 (7.7, 12.2) ng/mL and 7.5 (4.2, 12.0) ng/mL, respectively. There were no significant differences in age, menstrual cycle duration, timing of blood sample collection, and progesterone levels before menstruation between the two groups (Table 1).

**Table 1.** Demographic Characteristics of the Study Participants

	Total (n = 21)	PMS (+) (n = 7)	PMS (−) (n = 14)	*p*
Age (years)	22 (21, 22)	21 (20, 22)	22 (22, 22)	0.92
Menstrual cycle (days)	29.0 (28.0, 31.5)	28.5 (27.8, 30.5)	30.0 (28.0, 31.5)	0.82
Timing of blood collection before menstruation (days)	4.0 (5.0, 2.0)	2.0 (5.0, 1.8)	7.0 (5.5, 9.5)	0.97
Timing of blood collection after menstruation (days)	7.5 (6.0, 7.0)	7.5 (6.8, 9.0)	5.0 (5.5, 2.5)	0.42
Progesterone levels before menstruation (ng/mL)	7.9 (4.5, 12.5)	8.3 (7.7, 12.2)	7.5 (4.2, 12.0)	0.61

Data are presented median (IQR).

IQR, interquartile range; PMS, premenstrual syndrome.

### TNF-α levels

The TNF-α levels did not change significantly before and after menstruation (Table 2). In addition, there were no differences in the TNF-α levels before and after menstruation between the PMS (+) and PMS (−) groups.

**Table 2.** Tumor Necrosis Factor-Alpha and Interleukin-6 Levels in the Premenstrual Syndrome (+) and Premenstrual Syndrome (−) Groups Before and After Menstruation

	Before menstruation	After menstruation	*p*
TNF-α level (pg/mL)			
PMS (+) (*n* = 7)	11.3 (6.3, 8.3)	7.5 (5.8, 9.4)	1.000
PMS (−) (*n* = 14)	6.3 (4.7, 11.6)	6.7 (5.1, 9.0)	0.925
Total (*n* = 21)	7.1 (5.0, 10.0)	3.8 (5.0, 9.2)	0.931
*P*	0.823	0.881	
IL-6 level (pg/mL)			
PMS (+) (*n* = 7)	1.2 (0.4, 1.4)	0.3 (0.1, 0.9)	0.499
PMS (−) (*n* = 14)	0.9 (0.4, 1.2)	0.9 (0.6, 1.2)	0.594
Total (*n* = 21)	0.9 (0.3, 1.2)	0.7 (0.3, 1.2)	0.972
*P*	0.550	0.247	

Data are presented as median (IQR).

IL, interleukin; TNF, tumor necrosis factor.

### IL-6 levels

The IL-6 levels did not change significantly before and after menstruation (Table 2). In addition, there were no differences in the IL-6 levels before and after menstruation between the PMS (+) and PMS (−) groups.

### IL-10 levels

Overall, the IL-10 levels did not change significantly before and after menstruation (Table 3). In contrast, the IL-10 levels were significantly lower before than after menstruation in the PMS (−) group (*p* = 0.038) but not in the PMS (+) group. The IL-10 levels before menstruation were significantly higher in the PMS (+) group than in the PMS (−) group (*p* = 0.002). There were no differences in the IL-10 levels after menstruation between the PMS (+) and PMS (−) groups.

**Table 3.** Interleukin-10 and C-Reactive Protein Levels in the Premenstrual Syndrome (+) and Premenstrual Syndrome (−) Groups Before and After Menstruation

	Before menstruation	After menstruation	p^*^
IL-10 level (pg/mL)			
PMS (+) (*n* = 7)	16.4 (9.9, 19.0)	10.4 (6.4, 17.4)	1.000
PMS (−) (*n* = 14)	5.9 (3.5, 6.7)	8.0 (6.3, 9.6)	0.038^*^
Total (*n* = 21)	6.7 (4.3, 15.7)	8.2 (6.3, 10.6)	0.235
*p^#^*	0.002^*#*^	0.383	
CRP level (mg/L)			
PMS (+) (*n* = 7)	0.08 (0.1, 0.2)	0.11 (0.1, 0.3)	0.735
PMS (−) (*n* = 14)	0.19 (0.1, 1.3)	0.34 (0.2, 0.6)	0.861
Total (*n* = 21)	0.18 (0.1, 0.6)	0.24 (0.1, 0.4)	0.654
*p^#^*	0.025^*#*^	0.132	

Data are presented as median (IQR).

^*^ *p* < 0.05, before menstruation versus after menstruation; ^#^*p* < 0.05, PMS (+) versus PMS (−).

IL, interleukin; CRP, C-reactive protein.

### CRP levels

The CRP levels did not change significantly before and after menstruation in the PMS (+) and PMS (−) groups (Table 3). The CRP levels were significantly lower in the PMS (+) group than the PMS (−) group before menstruation (*p* = 0.025). However, the CRP levels were lower than the clinical reference of <3 mg/L.^23,24^

### CES-D scores

Overall, the CES-D scores did not change significantly before and after menstruation (Table 4). The CES-D scores tended to be higher before than after menstruation in the PMS (+) group (*p* = 0.063) but not in the PMS (−) group. The CES-D scores were higher in the PMS (+) group than in the PMS (−) group both before and after menstruation (*p* = 0.003 and *p* = 0.006, respectively).

**Table 4.** Center for Epidemiologic Studies Depression Scores in the Premenstrual Syndrome (+) and Premenstrual Syndrome (−) Groups Before and After Menstruation

CES-D score (point)	Before menstruation	After menstruation	*p*
PMS (+) (*n* = 7)	41 (28.0, 42.0)	26 (24.0, 35.0)	0.063
PMS (−) (*n* = 14)	11 (7.3, 16.8)	12 (8.0, 18.5)	0.834
Total (*n* = 21)	17 (9.0, 21.0)	16 (9.0, 25.0)	0.218
p^*^	0.003^*^	0.006^*^	

Data are presented as median (IQR).

^*^ *p* < 0.05, PMS 1+7 versus PMS 1−7.

CES-D, Center for Epidemiologic Studies Depression.

### Correlation

Overall, the correlation coefficient between the CES-D scores and IL-6 levels before menstruation was *r* = 0.49 (*p* < 0.001) and that between the CES-D scores and IL-10 levels after menstruation was *r* = 0.50 (*p* = 0.03). In the PMS (−) group, the correlation coefficient between the CES-D scores and IL-10 levels after menstruation was *r* = 0.83 (*p* < 0.001).

## Discussion

IL-10 has been shown to be an anti-inflammatory cytokine with antioxidant properties.^25^ In this study, the IL-10 levels before menstruation were significantly higher in the PMS (+) group than in the PMS (−) group. These results suggest that inflammation might have been greater in the PMS (+) group than in the PMS (−) group, and consequently, IL-10 production might have been induced in the PMS (+) group to counteract this high inflammation. In the PMS (−) group, the IL-10 levels were significantly lower before menstruation than after menstruation. Foster et al. analyzed IL-10 in young women aged 18–30 years without PMS and found that the IL-10 levels were significantly lower in the luteal phase than in the follicular phase.^26^ These findings are consistent with our results.

In healthy individuals, there is a regulated balance between pro- and anti-inflammatory cytokines. For example, IL-6 mediates the early phase of the inflammatory process and then induces the release of IL-10 that exerts immune-regulatory effects and reduces inflammation.^27^ In our study, there was no significant difference in IL-6. Samples were collected only once before and after menstruation; therefore, it might be necessary to increase the number of blood collections in a time-dependent manner (*e.g.*, the early luteal phase and late luteal phase). It has been reported that the cytokine balance could be shifted toward a proinflammatory status owing to elevated levels of proinflammatory cytokines, such as IL-6, and reduced levels of anti-inflammatory cytokines, such as IL-10.^28^ These findings indicate that such IL-10 levels might suppress the onset of PMS. This is consistent with the result of lower IL-10 levels in the luteal phase than in the follicular phase among healthy women.^26,29^ According to Foster et al., this fluctuation among healthy women who show increased IL-10 levels after menstruation is considered a complex inflammatory process involving inflammatory cytokines.^26^ In the clinical setting, anti-inflammatory agents have been found to provide relief from some symptoms related to PMS, such as abdominal pain, back pain, irritability, tiredness, and bloating.^30^

CRP, a general laboratory marker of immune activation and inflammation, was used as a nonspecific inflammatory biomarker. CRP levels before menstruation were significantly higher in the PMS (−) group than in the PMS (+) group. Despite this significant difference, the CRP levels were considered to have no clinical significance as individual levels were below the clinical reference.

In this study, there were no significant differences in TNF-α, IL-6, and CRP before and after menstruation in both groups. It is considered that increases in IL-10 levels possibly suppress the elevation of inflammatory markers, such as TNF-α, IL-6, and CRP.

Before and after menstruation, the CES-D scores were higher in the PMS (+) group than in the PMS (−) group. This finding suggests that depressive symptoms might have been more severe in participants with PMS than in those without PMS throughout the menstrual cycle. In addition, in the PMS (+) group, the CES-D scores tended to be higher (*i.e.*, more depressive) before than that after menstruation. This finding is consistent with the finding in a previous report showing that depressive symptoms worsened before menstruation in women with depression,^31^ and it might be due to an association between PMS and major depressive disorder.^32^ Furthermore, some criteria of PMDD scale, such as insomnia and depression, overlap with the CES-D scale. In this study, there were positive correlations between the CES-D scores and IL-6 levels before menstruation and the CES-D scores and IL-10 levels after menstruation. Several diseases, including mood and anxiety disorders, have been shown to be associated with inflammation.^33,34^ It has been suggested that proinflammatory cytokines, including IL-6, might contribute to depression in susceptible individuals with disorders involving chronic inflammation.^35,36^ Roque et al. reported that IL-10 levels are related to depression.^37^ Therefore, inflammation might underlie the pathogeneses of PMS and major depressive disorder.

The levels of inflammatory cytokines increase with age. However, the influence of age might not be significant in our study as our participants were young. In this study, there was no significant difference in progesterone levels before menstruation between the two groups. There are some previous studies that have assessed the associations between progesterone levels and the onset of PMS. In women with PMDD, peripheral levels of progesterone during the luteal phase were found to be either decreased^38,39^ or increased^40,41^ across different studies, whereas some other studies have found no significant changes in progesterone levels.^42,43^ The association between progesterone levels and the onset of PMS has yielded controversial results. This was a small preliminary study; future research should examine whether progesterone contributes to the onset of PMS using a larger study.

To our knowledge, this is the first study to compare the levels of inflammatory makers before and after menstruation in the same participants.

This study has several limitations. First, the sample size was small (*n* = 21). Second, PMS/PMDD was not diagnosed by prospective observation as has been recommended previously.^44^ Third, this study could not differentiate between premenstrual exacerbation and magnification of an underlying psychiatric or medical illness. In this study, we found for the first time that IL-10 level is increased in women with PMS during the luteal phase. However, as this was a small study, we considered that the power to detect true differences in some of the cytokine levels that we have measured might be low. In addition, multiple comparisons on our data might not be replicated in a larger study. However, considering the significance levels, there was a significant difference in IL-10 level in women with PMS compared those without PMS, and there was no overlap in the IQR values, indicating a clear difference. This would help establish a direction for further definitive studies and would be helpful to researchers interested in “PMS.” Therefore, we believe that it will be necessary to consider this aspect in a larger study in future. For the verification of our findings, larger studies with precise diagnosis of PMS and PMDD are desirable.

## Conclusions

The IL-10 levels before menstruation were higher in women with PMS than in those without PMS, and these levels might be related to PMS. There were no differences in other inflammatory markers between these patient groups, and there was no association among inflammatory markers. The response of IL-10 to IL-6 should be discussed as one possibility for IL-10 elevation in women with severe PMS. It is desirable to perform a large-scale study and increase the number of blood collection points for detailed investigation.

## Abbreviations Used

CES-D: Center for Epidemiologic Studies Depression
CRP: C-reactive protein
ELISA: enzyme-linked immunosorbent assay
IL: interleukin
IQR: interquartile range
PMDD: premenstrual dysphoric disorder
PMS: premenstrual syndrome
TNF: tumor necrosis factor
